# Identification of dynamic signatures associated with smoking‐related squamous cell lung cancer and chronic obstructive pulmonary disease

**DOI:** 10.1111/jcmm.14852

**Published:** 2019-12-12

**Authors:** Xiaoru Sun, Jingzhe Shang, Aiping Wu, Jingyan Xia, Feng Xu

**Affiliations:** ^1^ Department of Infectious Diseases The Second Affiliated Hospital Zhejiang University School of Medicine Hangzhou China; ^2^ Center of Systems Medicine Chinese Academy of Medical Science (CAMS) Suzhou Institute of System Medicine Suzhou China; ^3^ Department of Radiation Oncology The Second Affiliated Hospital Zhejiang University School of Medicine Hangzhou China

**Keywords:** chronic obstructive pulmonary disease, infection, oxidative stress, squamous cell lung cancer, TP53

## Abstract

Chronic obstructive pulmonary disease (COPD) is a risk factor for the development of lung cancer. The aim of this study was to identify early diagnosis biomarkers for lung squamous cell carcinoma (SQCC) in COPD patients and to determine the potential pathogenetic mechanisms. The GSE12472 data set was downloaded from the Gene Expression Omnibus database. Differentially co‐expressed links (DLs) and differentially expressed genes (DEGs) in both COPD and normal tissues, or in both SQCC + COPD and COPD samples were used to construct a dynamic network associated with high‐risk genes for the SQCC pathogenetic process. Enrichment analysis was performed based on Gene Ontology annotations and Kyoto Encyclopedia of Genes and Genomes pathway analysis. We used the gene expression data and the clinical information to identify the co‐expression modules based on weighted gene co‐expression network analysis (WGCNA). In total, 205 dynamic DEGs, 5034 DLs and one pathway including CDKN1A, TP53, RB1 and MYC were found to have correlations with the pathogenetic progress. The pathogenetic mechanisms shared by both SQCC and COPD are closely related to oxidative stress, the immune response and infection. WGCNA identified 11 co‐expression modules, where magenta and black were correlated with the “time to distant metastasis.” And the “surgery due to” was closely related to the brown and blue modules. In conclusion, a pathway that includes TP53, CDKN1A, RB1 and MYC may play a vital role in driving COPD towards SQCC. Inflammatory processes and the immune response participate in COPD‐related carcinogenesis.

## 1 INTRODUCTION

Chronic obstructive pulmonary disease (COPD) is characterized by progressive deterioration of lung function and incompletely reversible airflow obstruction in the lungs over time.^1^ COPD affects ~65 million people throughout the world^2^ and is a leading cause of years of life lost worldwide.^3^, ^4^ Lung cancer is a common and deadly malignancy worldwide^5^ and the leading cause of death due to cancer in almost every country.^6^, ^7^ Lung cancer accounts for 25% of all cancer deaths in the United States.^6^

COPD and lung cancer are two of the most important smoking‐related diseases worldwide. Nearly 1 billion people around the world have the habit of smoking. Smoking is the leading cause of COPD, which is considered a risk factor for lung cancer,^8^, ^9^, ^10^, ^11^, ^12^ particularly squamous cell carcinoma (SQCC).^13^ SQCC accounts for 20%‐30% of non–small‐cell lung cancer (NSCLC) cases, and it is strongly associated with a history of cigarette smoking. Lung cancer is up to five times more likely to occur in smokers with airflow obstruction than those with normal lung function.^14^ About 50%‐70% of the smokers with lung cancer have pre‐existing COPD prior to their cancer diagnosis.^15^, ^16^ The annual incidence of lung cancer arising in patients with COPD has been reported as 0.8%‐1.7%.^17^, ^18^ Therefore, smoking, COPD and SQCC are closely related. However, the mechanisms that allow COPD to increase the risk of developing SQCC as well as the influence of COPD on the prognosis of SQCC in patients who are former or current smokers are not clear although it is accepted that chronic immune inflammation, premature ageing in the lungs,^19^ telomere shortening,^20^, ^21^ systemic inflammation, oxidative stress and lung repair mechanisms 22 probably play roles in the pathogenesis of lung cancer. However, the mechanisms that drive COPD to develop SQCC are still unknown. Therefore, a comprehensive analysis is required of the molecular signatures of the pathogenic processes in both COPD and SQCC.

In this study, we aimed: (a) to determine the shared mechanisms that play key roles in the pathogenesis of SQCC from COPD in smokers and (b) to identify dynamic biomarkers and potential therapy targets for SQCC in COPD patients.

## 2 MATERIALS AND METHODS

### 2.1 Differential gene expression analysis and construction of the network associated with differentially expressed genes (DEGs)

The data of GSE12472 23 were obtained from NCBI Gene Expression Omnibus (GEO) (://www.ncbi.nlm.nih.gov/geo/). The data set comprised three groups: 10 laser microdissected histologically normal bronchial epithelium samples (“normal”), 18 microdissected histologically bronchial epithelium samples from patients with COPD (“COPD”) and 18 centrally located primary SQCC tissue samples obtained from patients with COPD (“SQCC + COPD”). All of the samples were derived from people who were smokers or ex‐smokers. No statistically significant differences in age, sex or the history of smoking were found among the three groups. After background correction, we average the expression among probes that map to the same gene, and the average value of expression was transformed into a normalized expression value using the *Z*‐score. DEG 1 between the COPD group and normal group, and DEG 2 between the SQCC + COPD group and COPD group were compared with Student's *t* test. The detailed criterion for DEGs was defined as FDR (false discovery rate) <0.05. Dynamic DEGs were defined as the overlap between DEG 1 and DEG 2. The protein‐protein interaction (PPI) network associated with the dynamic DEGs (FDR < 0.05) was constructed using STRING (http: http://www.string-db.org/), and highly correlated genes/proteins (confidence score > 0.4) were selected as inclusion criteria.

### 2.2 Differential expression analysis of gene pairs

We calculated Pearson's correlation coefficient (PCC) for each pair of genes from the three groups using the expression profiles in the data set. The differential PCC (d‐PCC) 1 between the COPD group and normal group, and d‐PCC 2 between the SQCC + COPD group and COPD group were calculated. The gene pairs with absolute d‐PCC values ranging from 0.8 to 2 were selected as the differentially co‐expressed links (DLs). The dynamic DLs were calculated as the overlap between d‐PCC 1 and d‐PCC 2.

### 2.3 Dynamic protein‐protein interaction (PPI) network construction

The PPI data from the Biological General Repository for Interaction Datasets (BioGRID; http://www.thebiogrid.org), Human Protein Reference Database (HPRD; http://www.hprd.org), TRED (http://rulai.cshl.edu/cgi-bin/TRED) and KEGG (http://www.genome.jp/kegg) were merged into the background PPI network. Then, the DLs were then mapped onto the background PPI network. The interconnection between two genes was assessed based on the degree of their shared neighbours across the PPI. The network diagram of PPI was visualized with Cytoscape (version 3.6.0).

### 2.4 Gene Ontology (GO) and Kyoto Encyclopedia of Genes and Genomes (KEGG) pathway enrichment

GO functional annotation and enrichment analysis as well as KEGG pathway enrichment for the PPI were accomplished using R package (clusterprofiler package). False discovery rate (FDR) was calculated for *p*‐value correction. A KEGG pathway with a BH‐corrected *P* < .05 was considered to be significantly enriched.

### 2.5 Regulatory pathways

To establish the most statistically significant biological pathways of the PPI, Ingenuity Pathway Analysis (IPA) software (IPA®, QIAGEN) was used, for network associations and post‐transcriptional targets regulation.

### 2.6 Construction of Weighted gene co‐expression network analysis (WGCNA)

The data of GSE12472 were used for WGCNA under R package WGCNA, and the power parameter was pre‐calculated by the pickSoftThreshold function. An appropriate soft‐thresholding power was selected according to standard scale‐free distribution. The modules were identified with a dynamic tree‐cutting algorithm. The intramodular connectivity was used to define the most highly connected hub gene in a module. The co‐expression network of genes within the pathological stage‐related module was visualized with Cytoscape software.

## 3 RESULTS

### 3.1 DEGs

**Figure 1:**
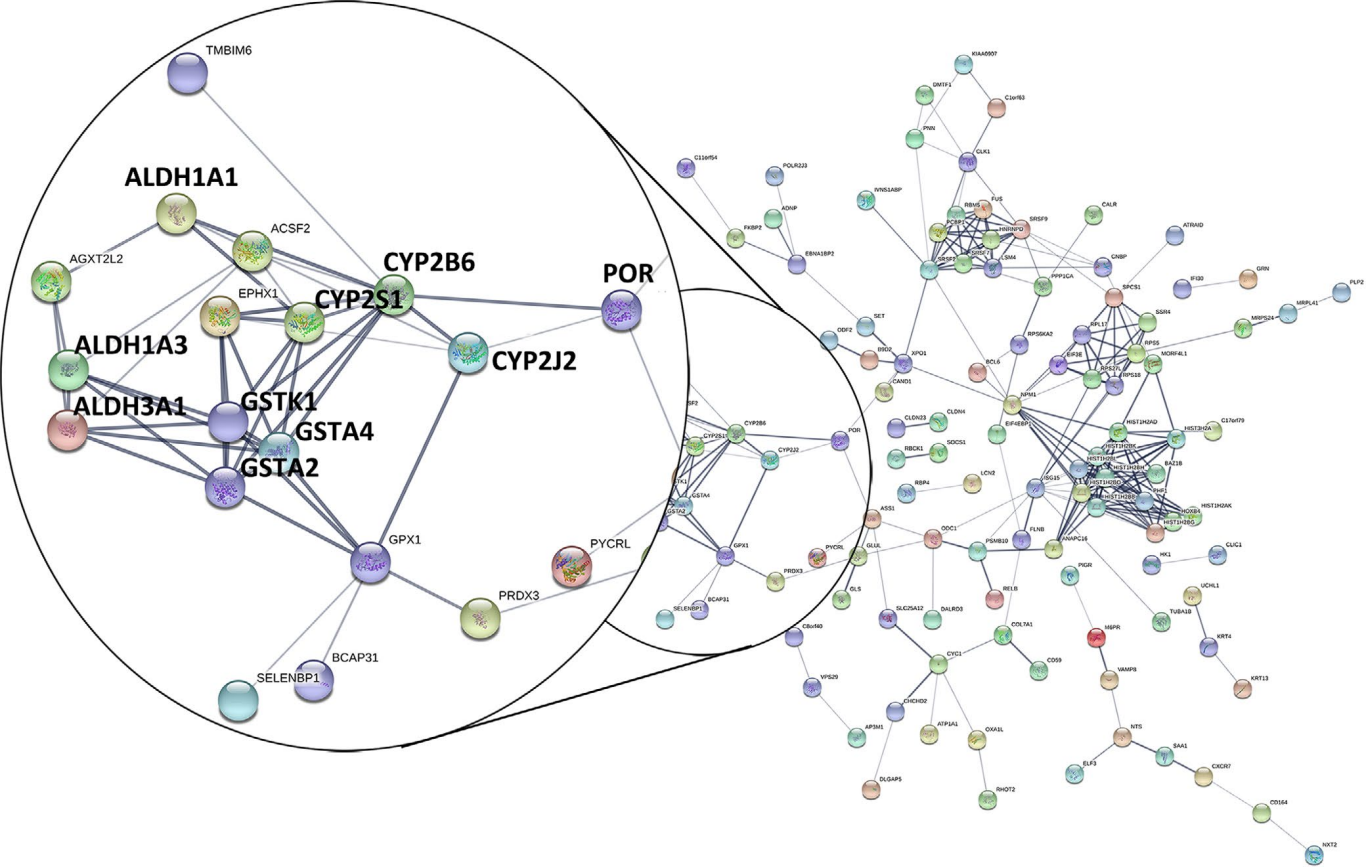
Protein‐protein interaction (PPI) network of dynamic differentially expressed genes (DEGs) (FDR < 0.05) constructed by STRING. Interactions at medium confidence (score > 0.4) and evidence from experiments, database searches and text mining were considered. Black circle shows the zoom‐in the significant module of the PPI. Nodes with no or scattered interactions were excluded

**Figure 2:**
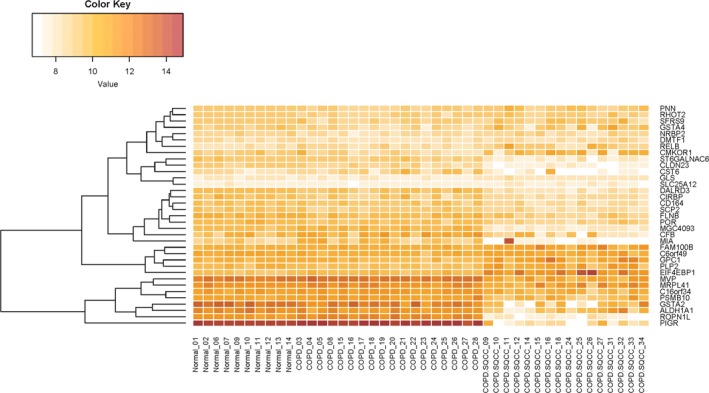
Hierarchical clustering analysis of DEGs. Heatmap of the top 35 dynamic DEGs (FDR < 0.01). The red colour in the heatmap denotes higher gene expression, and the white colour in the heatmap denotes the lower gene expression. Target gene symbols for the top 35 DEGs are involved

In total, 205 dynamic DEGs met the criterion of a FDR < 0.05 for both DEGs. The PPI network for the 205 dynamic DEGs was constructed using String (Figure 1), and 35 genes met the criterion of a FDR < 0.01 (Table S1 and Figure 2). The PPI network contained four sub‐networks. One of the sub‐networks included many dynamic DEGs (FDR < 0.01), such as ALDH1A1, GSTA2, GSTA4 and POR.

### 3.2 Related pathways involving DEGs

**Figure 3:**
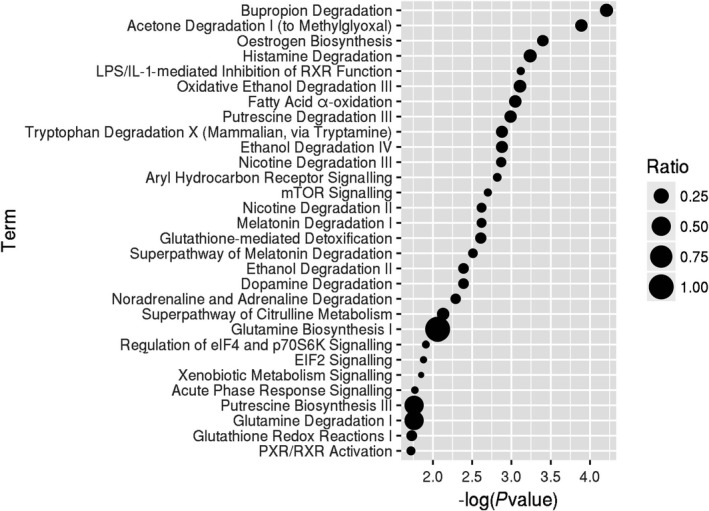
Scatter plot of canonical pathways based on ingenuity pathway analysis (IPA). Canonical pathway of the dynamic DEG. Ratio is the ratio of numbers of DEGs annotated in this pathway term to the numbers of all genes annotated in this pathway term. The data presented are log‐transformed *P*‐value (FDR corrected) of pathways found to be enriched in the tested group of genes

The dynamic DEGs (FDR < 0.05) were used to understand the enriched functions. We analysed the canonical pathways based on IPA. Seven pathways were significantly enriched (*P*‐value < .05) comprising bupropion degradation, acetone degradation I (to methylglyoxal), oestrogen biosynthesis, histamine degradation, LPS/IL‐1‐mediated inhibition of RXR function, oxidative ethanol degradation III and fatty acid α‐oxidation (Figure 3). The three main pathways involved in drug and xenobiotic metabolism included genes encoding cytochrome p450 enzymes. The enzymatic activation of the potent lung carcinogenic tobacco‐specific nitrosamines (TSNA) and 4‐(methylnitrosamino)‐1‐(3‐pyridyl)‐1‐butanone (NNK) has been found related to NNK‐induced lung tumours,^24^ and the CYP2B6 enzyme has a high affinity for NNK, that is, roughly 10 times higher compared with CYP2A6.^25^ Moreover, smoking was found to accelerate the production of carcinogenic oestrogenic metabolites 4‐hydroxy (4‐OHEs) metabolites in the lungs.^26^ Cytochrome P450 1b1 (CYP1B1) probably participated in the increased susceptibility of the female gender to tobacco.^27^ NNK was shown to induce ERα by CYP1B1 activation,^28^ and anti‐oestrogens inhibited NNK‐induced murine lung carcinogenesis.^29^

### 3.3 Differential co‐expression pairs and the dynamic PPI network

**Figure 4:**
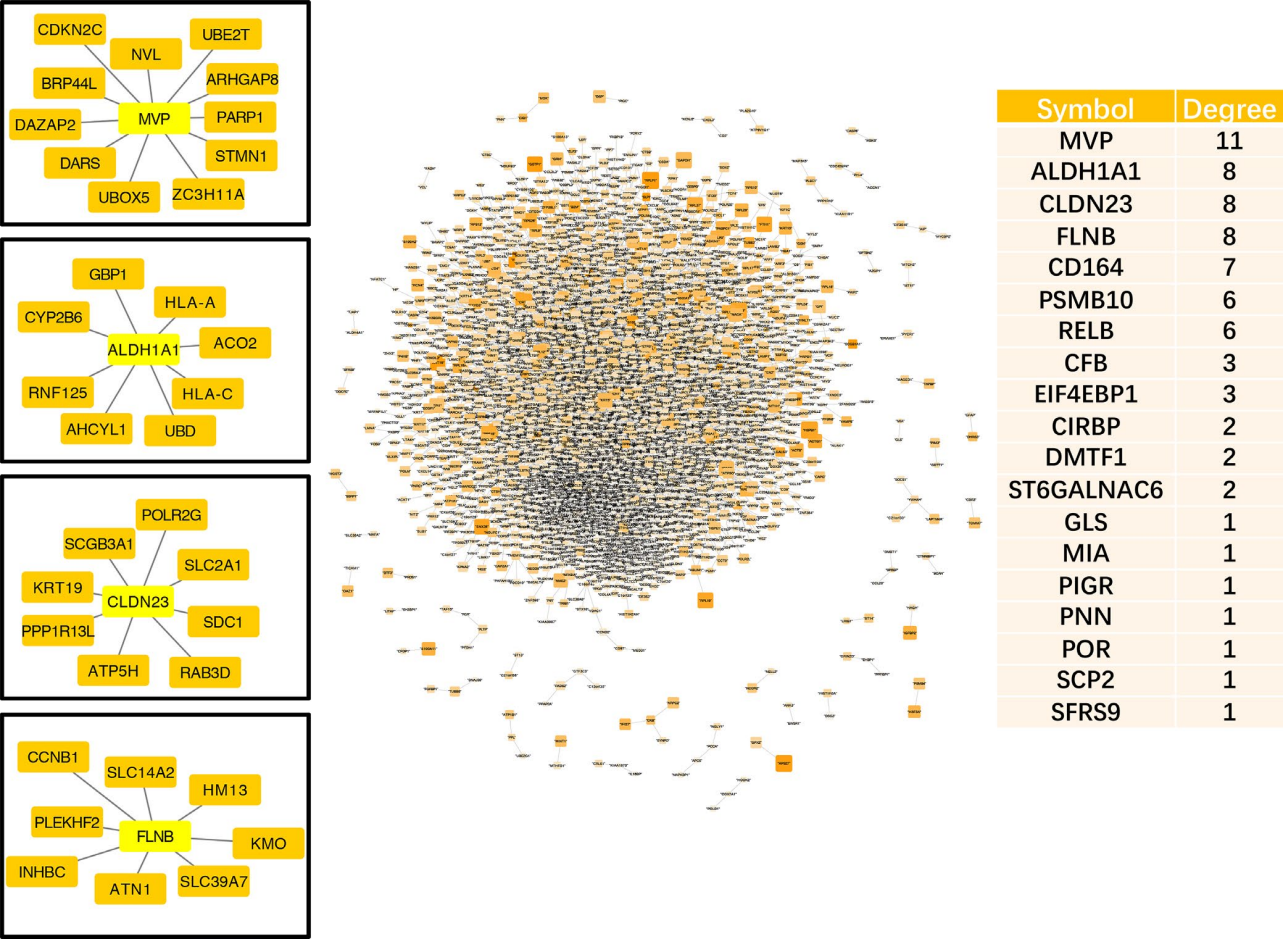
The human PPI network of squamous cell lung cancer (SQCC) pathogenic process‐associated genes. All the DEGs and DLs were assembled based on the d‐PPC. The network was visualized using the Cytoscape program. The expression of chronic obstructive pulmonary disease (COPD) is represented by the colour of the circle. Orange represents a higher level of expression, and white represents lower expression. The expression of SQCC + COPD is represented by the size of the circle. The right table is the degree (number of neighbours) of the dynamic DEGs (FDR < 0.01) in the PPI. Among the DEGs, the network of ALDH1A1, MVP, CLDN23 and FLNB was displayed

Pearson's correlation coefficients (PCCs) were calculated to select the DLs by ensuring that both absolute d‐PCC values ranged from 0.8 to 2, that is, the d‐PCC for COPD compared with the normal group and the d‐PCC for SQCC + COPD compared with the COPD group. The DLs and the dynamic DEGs (FDR < 0.01) were then mapped to the known PPI network. Finally, we constructed the dynamic PPI network comprising 5034 DLs. In the dynamic PPI, we also calculated the degree of the dynamic DEGs (FDR < 0.01). PPI network analysis showed that the significant hub proteins included MVP (Degree = 11), ALDH1A1 (Degree = 8), CLDN23 (Degree = 8) and FLNB (Degree = 8) (Figure 4).

### 3.4 GO and KEGG pathway analysis of the dynamic PPI network

**Figure 5:**
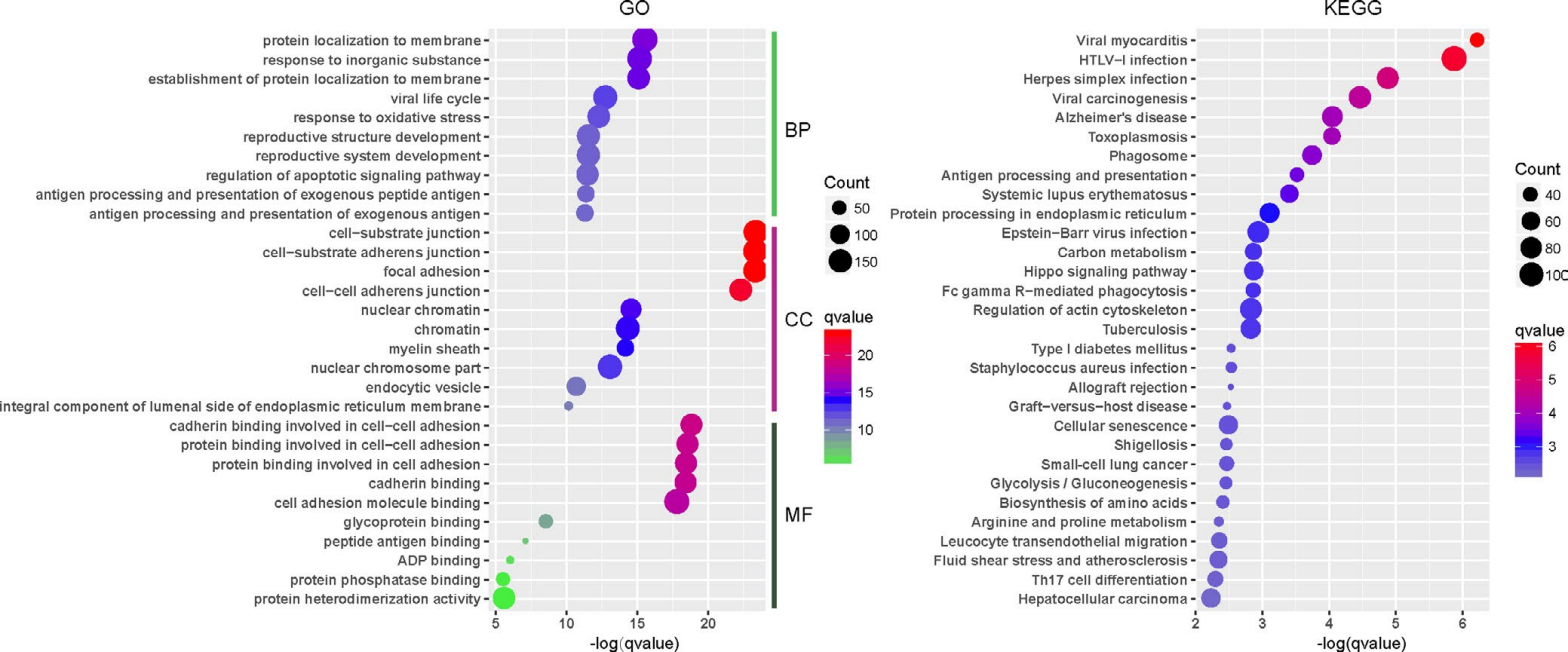
GO and KEGG enrichment scatter plot of the PPI network. The *y*‐axis shows significantly enriched GO and pathway terms relative to the network, and the *x*‐axis shows the enrichment scores of these terms. Dot size represents the number of genes, and the colour indicates the *q*‐value. BP, biological process; CC, cellular component; GO, gene ontology; MF, molecular function

Proteins work together to exert certain functions. The alterations in their networks usually suggest the development of disease. Therefore, the interaction network was established to identify the underlying molecular mechanisms. Based on the KEGG pathways and GO annotations for the PPI network, analyses were performed with the clusterprofiler package to identify the associated biological functions (Figure 5).

GO enrichment analysis was also conducted for the PPI network. In total, 62 molecular function terms were significantly over‐represented by using a *P*‐value < 0.01 as the cut‐off. The significantly altered function terms comprised: (a) cadherin binding involved in cell‐cell adhesion; (b) protein binding involved in cell‐cell adhesion; (c) protein binding involved in cell adhesion; (d) cadherin binding; (e) cell adhesion molecule binding; (f) glycoprotein binding;(g) peptide antigen binding; (h) ADP binding; (i) protein phosphatase binding; and (j) protein heterodimerization activity.

In total, 1090 biological process terms met the criterion of a *P*‐value < 0.01. The top 10 terms comprised: (a) protein localization to membrane; (b) response to inorganic substance; (c) establishment of protein localization to membrane; (d) viral life cycle; (e) response to oxidative stress; (f) reproductive structure development; (g) reproductive system development; (h) regulation of apoptotic signalling pathway; (i) antigen processing and presentation of exogenous peptide antigen; and (j) antigen processing and presentation of exogenous antigen.

In addition, 30 terms that satisfied the cut‐off (*P*‐value < 0.01) were obtained by KEGG enrichment analysis. As shown in Figure 5, the network was enriched for the following pathways (top 10): (a) viral myocarditis; (b) HTLV‐I infection; (c) herpes simplex infection; (d) viral carcinogenesis; (e) Alzheimer's disease; (f) toxoplasmosis; (g) phagosome; (h) antigen processing and presentation; (i) systemic lupus erythematosus; and (j) protein processing in endoplasmic reticulum.

### 3.5 Upstream analysis of DEGs and network

The upstream regulators of key genes are very important for understanding pathogenetic mechanisms. Using IPA software, we found 2889 upstream factors in the PPI, including drug, cytokine and transcription factors, as follows: (a) dexamethasone (*P* = 7.66E‐68); (b) TP53 (*P* = 1.26E‐58) (Figure S1); (c) TGFB1 (*P* = 1.52E‐57); (d) beta‐estradiol (*P* = 5.46E‐57); (e) MYC (*P* = 1.81E‐53); (f) TNF (*P* = 4.52E‐52); (g) lipopolysaccharide (*P* = 5.09E‐50); (h) tretinoin (*P* = 2E‐49); (i) IFNG *(P* = 2.52E‐45); and (j) ESR1 (*P* = 4.34E‐44). Moreover, some factors were closely related to SQCC as follows: (13) IL1B (*P* = 3.48E‐37); (21) PTEN (*P* = 9.64E‐28); (48) NFE2L2 (*P* = 1.79E‐21); and (257) PI3K (complex) (*P* = 2.38E‐09). In particular, the TP53, NFE2L2 and PI3K pathways had strong relationships with SQCC.

### 3.6 Detection of clinically key modules

**Figure 6:**
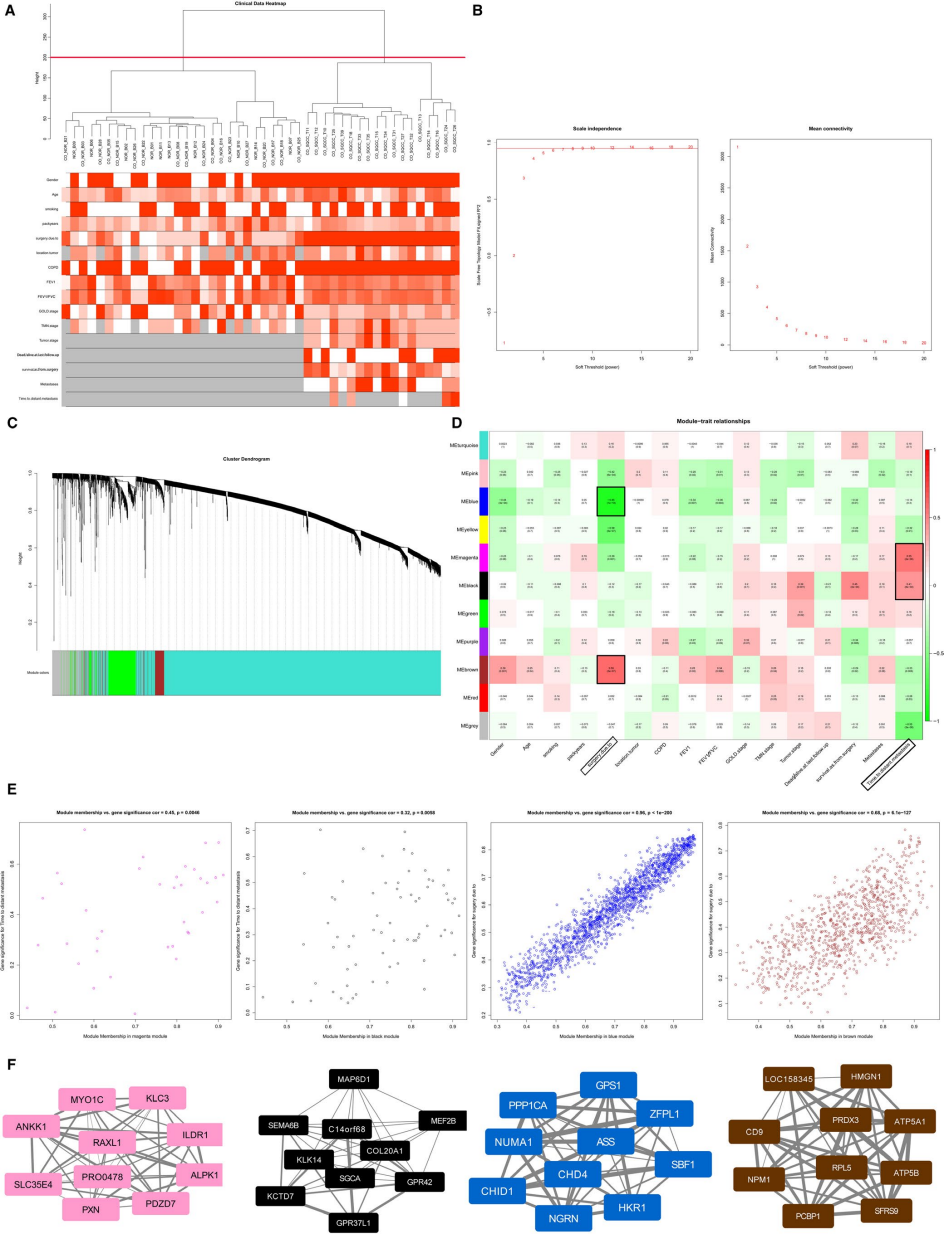
Weighted gene co‐expression network analysis. A, Sample clustering with no evident outliers. B, Analysis of the network topology showed that it satisfied the scale‐free topology threshold of 0.9 when β = 8. The left panel shows analysis of the scale‐free fit index for various soft‐thresholding powers (β). The right panel shows the mean connectivity analysis of various soft‐thresholding powers. C, Clustering dendrograms of genes based on dissimilarity topological overlap and module colours. The branches of the cluster dendrogram correspond to the 11 different gene modules. Each piece of the leaves on the cluster dendrogram corresponds to a gene. D, Correlations between the gene modules and clinical traits. E, Scatter plots of gene significance (GS) for metastasis vs. module membership (MM) in the magenta, black, blue and brown modules. F, Top 10 hub genes in the magenta, black, blue and brown modules

The R package for WGCNA was applied to the data for GSE12472 (Figure 6). There were no obvious outliers according to the sample clustering results (Figure 6A), and the power of β = 8 (scale‐free *R* ^2^ = 0.9) was selected as the soft‐threshold power to ensure a scale‐free network (Figure 6B). A total of seven modules that are highly co‐expressed were identified (Figure 6C).

According to the module‐feature relationships, we found that “time to distant metastasis” was strongly related to the magenta module (*r* = 0.55, *P* = 2E‐6) and “time to distant metastasis” was as well as highly associated with the black module (*r* = 0.45, *P* = 2E‐4) based on PCC analysis. And the highest association was found between the blue module and “surgery due to” (*r* = −0.85, *P* = 1E‐18). The “surgery due to” was also closely related to the brown module (*r* = 0.59, *P* = 3E‐7; Figure 6D). Scatter plots of gene significance vs. module membership for these modules are shown in the Figure 6E. Figure 6F illustrates the correlation between module membership and gene significance in the magenta, black, blue and brown modules, respectively.

## 4 DISCUSSION

Increasing evidence indicates that COPD may increase the risk of lung cancer, particularly SQCC.^13^ COPD and SQCC share tobacco smoking as a common risk factor, and they may also share similar pathogenetic mechanisms. Unfortunately, the pathogenetic mechanism remains elusive and little information is available regarding possible biomarkers and therapeutic targets. SQCC is much harder to detect than adenocarcinomas in terms of the dominant mutation related to tumour progression. The dominant mutations found frequently in lung adenocarcinomas, such as EGFR, ALK and KRAS mutations, are uncommon in SQCC.^30^

Different from the published article which reveals static analyses of the differences between SQCC patients with and without COPD,^23^ our study focused on the dynamic analysis of the pathogenetic process from normal to COPD, and with the further progression to SQCC. In this study, we found 205 dynamic DEGs (FDR < 0.05) that had associations with the progress from normal to COPD and finally to SQCC. The top canonical pathways for the 205 dynamic DEGs comprised bupropion degradation (POR, CYP2J2, CYP2B6 and CYP2S1), acetone degradation I (to methylglyoxal) (POR, CYP2J2, CYP2B6 and CYP2S1), oestrogen biosynthesis (POR, CYP2J2, CYP2B6 and CYP2S1), histamine degradation (ALDH1A1, ALDH1A3 and ALDH3A1), LPS/IL‐1‐mediated inhibition of RXR function (GSTA2, ALDH1A1, ALDH1A3, GSTA4, XPO1, CYP2B6, ALDH3A1 and GSTK1), oxidative ethanol degradation III (ALDH1A1, ALDH1A3 and ALDH3A1) and fatty acid α‐oxidation (ALDH1A1, ALDH1A3 and ALDH3A1). In particular, GSTA2, ALDH1A1, POR and GSTA4 were involved with the dynamic DEGs (FDR < 0.01) and they were included in a PPI sub‐network (Figure 1). The expression levels of the first three DEGs were down‐regulated during the course of the disease. GSTA4 was down‐regulated in COPD samples compared with the normal samples and up‐regulated in SQCC + COPD compared with the COPD samples. GSTA2 functions in the detoxification of electrophilic compounds, including carcinogens, therapeutic drugs, environmental toxins and products of the oxidative stress, by conjugating with glutathione (GSH). ALDH1A1 is the next enzyme after alcohol dehydrogenase in the major alcohol metabolism pathway, and it is related to oxidoreductase and acyl‐CoA dehydrogenase activities. The diseases associated with ALDH1A1 include lung adenoma. POR is an essential enzyme for multiple metabolic processes, particularly the reactions catalysed by cytochrome P450 proteins to metabolize steroid hormones, drugs and xenobiotics.

Interestingly, the down‐regulated genes are part of the GSH metabolism pathway and they are related to redox reactions. The GSH antioxidant system is an important defensive system in the body, which is crucial for protection against oxidative stress‐induced liver injury. In the lung, smoking exposure also elicits a powerful GSH adaptive response.^31^ Glutathione‐S‐transferases (GSTs) can promote the synthesis of GSH. In COPD patients, decreased serum GSH contents and reduced GST serum activities were found, which lead to an increased susceptibility to oxidative stress.^32^ The progression of COPD is partly driven by oxidative stress within the lungs. The elevation of tumour tissue GSH in SQCC 33 increases the antioxidant capacity and the resistance to oxidative stress as observed in many cancer cells.^34^ The intracellular GSH content has a decisive effect on anticancer drug‐induced apoptosis.^34^ GSH depletion can enhance cytotoxicity and decrease resistance to chemotherapy.^35^ However, the protein levels of several antioxidant genes, including GSTA, which are involved with GSH homeostasis in the lung, do not increase in a linear manner as the disease progresses and they may even be down‐regulated as the disease progresses to the terminal stages.^36^ Consequently, the down‐regulated antioxidant genes influence GSH homeostasis and limit the functions of GSH. In addition, the common upstream regulator of GSTA2, GSTA4^37^ and ALDH1A1^38^ is Nrf2, and Keap1/Nrf2/Cullin3 pathway alterations occur in a third of SQCC cases according to The Cancer Genome Atlas (TCGA) discoveries. Moreover, in our study, ALDH1A1 was the second‐highest ranking dynamic DEG, with a degree of eight across the dynamic PPI network. In general, ALDH1A1 has prognostic value as a marker in patients with head and neck squamous cell carcinoma.^39^ Recent studies have demonstrated that ALDH1A1 expression is reduced in 25.5% (11/43) of SQCC^40^ cases, and the loss of ALDH1A1 expression may promote carcinogenesis, especially in smoking patients. Consequently, ALDH1A1 is the most likely candidate for use as a biomarker of disease progression.

We conducted dynamic PPI analysis in order to further explore the mechanisms related to disease progression. According to the KEGG and GO enrichment results, we found that 30 pathways were involved with the various biological processes enriched in the dynamic PPI network. The top 10 biological processes were response to inorganic substance, response to oxidative stress, reproductive structure development, regulation of apoptotic signalling pathway, antigen processing and presentation of exogenous antigen. The top pathways included viral myocarditis, HTLV‐I infection, herpes simplex infection, viral carcinogenesis, Alzheimer's disease, toxoplasmosis, phagosome and antigen processing and presentation.

GO analysis again demonstrated that oxidative stress has a key role in disease progression. Inorganic substances may comprise oxidants/free radicals related to smoking exposure.^41^ Oxidants/free radicals are considered to be responsible for oxidative stress. Excessive oxidative stress can initiate lung tumorigenesis, and it has been shown to have a significant role in DNA damage in the lungs during COPD.^42^ In addition, oxidative stress can induce oncogenic lipid peroxides, inactivate defensive mechanisms and lead to changes in the extracellular matrix. Moreover, some upstream regulators, including NRF2 and PTEN signalling, were found to have relationships with the oxidative stress response, thereby supporting our previous conclusion. Exposure to cigarette smoke exposure has an important role in the SQCC disease process.

The KEGG analysis results indicated that COPD may be caused by an aberrant inflammatory cascade with exaggerated cellular immunity and oxidative stress damage to lung tissue. Bacterial infection is often accompanied by a strong inflammatory response, with the release of proinflammatory mediators and recruitment of large numbers of neutrophils to the lung.^43^ This response is important for the control of infection,^44^ but excessive neutrophilic inflammation can also lead to ROS release. Therefore, the continuous activation of neutrophils can promote the accumulation of DNA damage, thereby leading to degranulation and the subsequent proteases release, involving neutrophil elastase from azurophilic granules.^45^ Viral infection can directly lead to the result of neutrophil necrosis and azurophilic granular release in COPD.^46^, ^47^ A deficiency of α1‐AT, an anti‐proteinase, is the only known heritable defect that causes an accelerated emphysematic phenotype, and it is significant that a low α1‐AT level is related to the possibility of lung cancer development.^48^ Neutrophil elastase activity is essential for promoting tumour angiogenesis and proliferation, 49 and it regulates the activity of the PIK3CA/Akt pathway by degrading its binding partner, IRS1 to accelerate lung cancer growth.^50^ Therefore, excessive neutrophil degranulation can promote tumour initiation by releasing ROS to contribute to DNA damage, and its proteolytic content may also activate the proliferation, and migration of tumours, as well as angiogenesis.

Pathway analysis also indicated that the progress of the disease is strongly associated with the immune response. In COPD patients, the supportive factors that are released in the tumour microenvironment can shift the airway macrophages into M2 activation.^51^ Adaptive immunity is also compromised in the patients with COPD, which may be related to the increasing number of exhausted T cells which are unable to respond effectively to respiratory infections effectively. In COPD, T cell anergy potentially results in tumour escape by suppressing the clearance of tumour cells by cytotoxic T cells. CTLA‐4 and PD‐1 inhibitors can be used to stimulate the T cells in COPD to mitigate the risk of disease progression and lung cancer, but the activation of cytotoxic T cells can influence the development of emphysema via the apoptosis of structural cells. The concentration of PD‐1^+^ exhausted effector T cells in the serum is increased in COPD,^52^ which may mitigate the deficiency of anti‐infection functions, whereas cancer immune surveillance could also be compromised.

**Figure 7:**
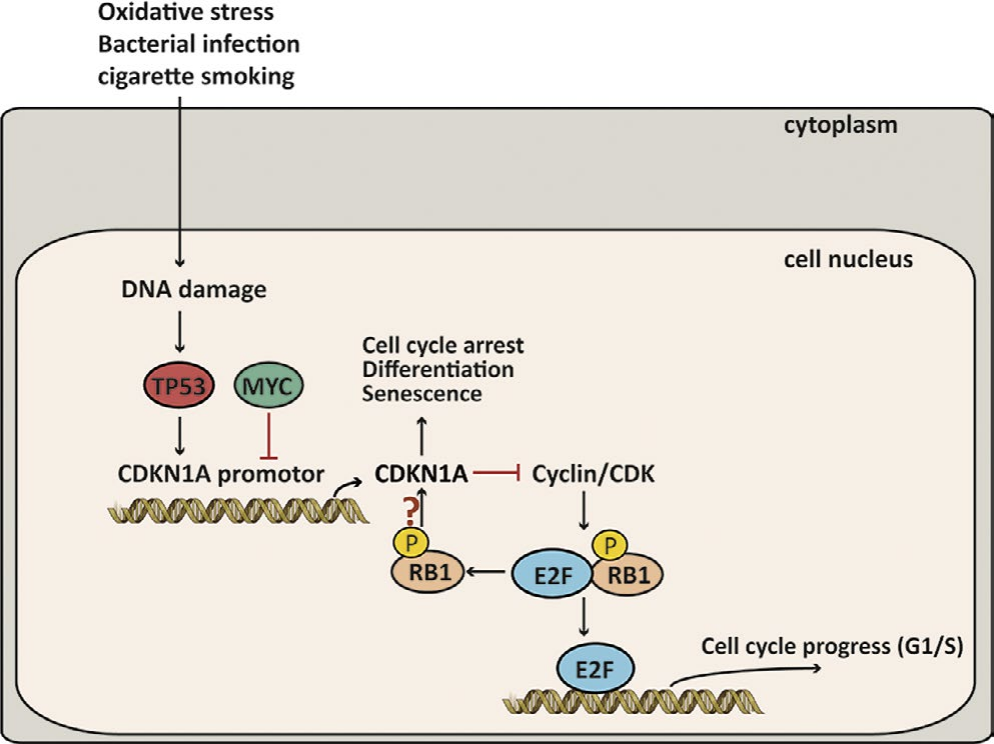
Hypothetical pathway related to the disease pathogenesis process. The hypothetical pathway may include down‐regulated RB1 and TP53 and up‐regulated MYC, which together repress the activation of CDKN1A. This further prevents cell cycle arrest and apoptosis, in turns driving COPD towards SQCC. Meanwhile, the down‐regulated RB1 hardly promotes cell cycle progress

**Figure 8:**
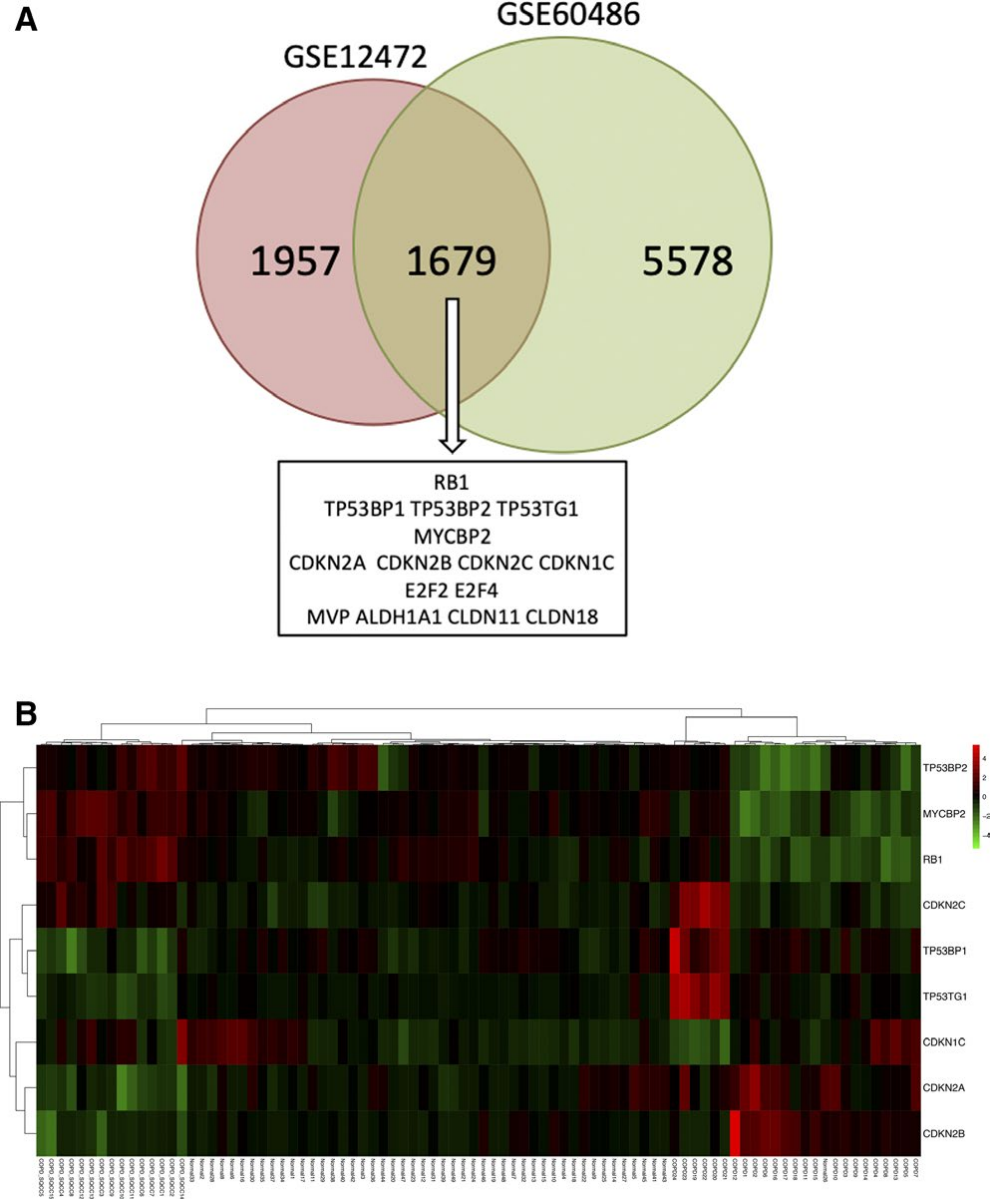
Validation in hub genes and the hypothesized pathway‐related genes in GSE60486. A, Identification of common genes between the PPI network of GSE12472 and the PPI network of GSE60486 by overlapping them. The hub genes and pathway‐related genes were also in GSE60486. B, Heatmap hierarchical clustering showed the selected genes clustering in the three stages of the SQCC pathogenetic progress

Upstream analysis was performed based on the dynamic PPI network, and 2888 upstream regulators were identified. The top two regulators were dexamethasone and TP53, and PTEN and Nrf2 were also included in the top 50 upstream regulators, while PI3K/Akt ranked 257th (*P* = 2.38E‐09). TP53 is a stress response gene that activates the transcription of numerous downstream genes in response to genotoxic stress, oncogenic signalling, DNA damage and cellular injury.^53^ The frequency of TP53 mutations is the highest in SQCC and lower in adenocarcinomas among NSCLC tumour samples.^54^, ^55^ In our study, we found that dynamic DEGs including MVP and SCP2 were involved in the downstream regulation of TP53. In addition, a pathway downstream of TP53 involving CDKN1A, MYC and RB1 among the DLs was related to the pathogenetic process. We found that TP53, CDKN1A and RB1 were down‐regulated continuously in the disease process, whereas MYC was up‐regulated from the COPD group to the COPD + SQCC group. TP53, RB1 and MYC are usually considered to be mutated in small‐cell lung cancer (SCLC).^56^, ^57^ Because of the opposite effect of MYC and TP53 in the CDKN1A regulation, the inactivation of TP53 may interfere with MYC‐based targeted therapies. MYC blocks the function of p21 in many situations,^58^ whereas TP53 is a transcription activation factor for CDKN1A.^59^ As a result, the up‐regulation of MYC would allow the TP53‐dependent inactivation of CDKN1A, which can then repress the cell cycle arrest and apoptosis. In addition, the inhibition of RB1 is known to prevent MYC inhibition from inducing apoptosis in TP53‐inactivated melanoma cells.^60^ Therefore, it is likely that the down‐regulated RB1 and TP53 as well as the up‐regulated MYC would together repress the activation of CDKN1A to further prevent cell cycle arrest and apoptosis, thereby leading COPD to progress towards SQCC (Figure 7). In order to validate the hypothesized pathway, we selected data from another data set GSE60486 of GEO database. We selected 49 normal samples, 24 COPD samples and 15 SQCC + COPD samples. A new dynamic network associated with the high‐risk genes for the SQCC pathogenetic process was constructed using the method described above. The new network included 7257 genes, and we overlapped the new network with the GSE12472 network in a Venn diagram and found that 1679 genes were shared, including the hub genes and the genes in the pathways (Figure 8A). Furthermore, we selected nine genes comprising RB1; TP53‐related genes TP53BP1, TP53BP2 and TP53TG1; CDKN1A‐related genes CDKN1C, CDKN2A, CDKN2B and CDKN2C; and MYC binding protein 2 (MYCBP2). The penal of the genes clustered in the three stages of the SQCC pathogenetic progress (Figure 8B), thereby demonstrating that the selected genes in the assumed pathway could potentially be used as a panel of novel biomarkers for predicting COPD‐related carcinogenesis. Further clinical studies are needed to clarify the connections among these genes in SQCC cells.

## 5 CONCLUSION

In this study, we constructed a dynamic PPI network to identify the dynamic genes/pathways associated with the pathogenetic process from normal to COPD, and with the further progression to SQCC. Based on t tests, 205 genes were selected as dynamic DEGs. In addition, we hypothesized that a pathway including TP53, CDKN1A, RB1 and MYC may play vital roles in the disease pathogenetic process. Moreover, inflammatory processes may play central roles in COPD carcinogenesis. However, future studies are required to further validate these DEGs in lung tissues from non‐smoking patients with COPD and SQCC + COPD. Some of the main DEGs identified in this study, including ALDH1A1, may be used to predict the risk of developing smoking‐related SQCC from COPD. More clinical studies are also required to validate these genes and the hypothetical pathway in SQCC and COPD.

## CONFLICT OF INTERESTS

The authors declare that they have no conflicts of interest.

## AUTHORS’ CONTRIBUTIONS

Feng Xu and Jingyan Xia designed the work. Xiaoru Sun and Jingzhe Shang analysed the data with the guidance of Feng Xu and Aiping Wu. Xiaoru Sun and Feng Xu prepared the manuscript. All authors approved the final version of the manuscript.
