# Supplementary figures and images for: Identification of dynamic signatures associated with smoking‐related squamous cell lung cancer and chronic obstructive pulmonary disease
Source: J Cell Mol Med. 2019 Dec 12;24(2):1614–25. doi: 10.1111/jcmm.14852 (PMC6991676; doi:10.1111/jcmm.14852)

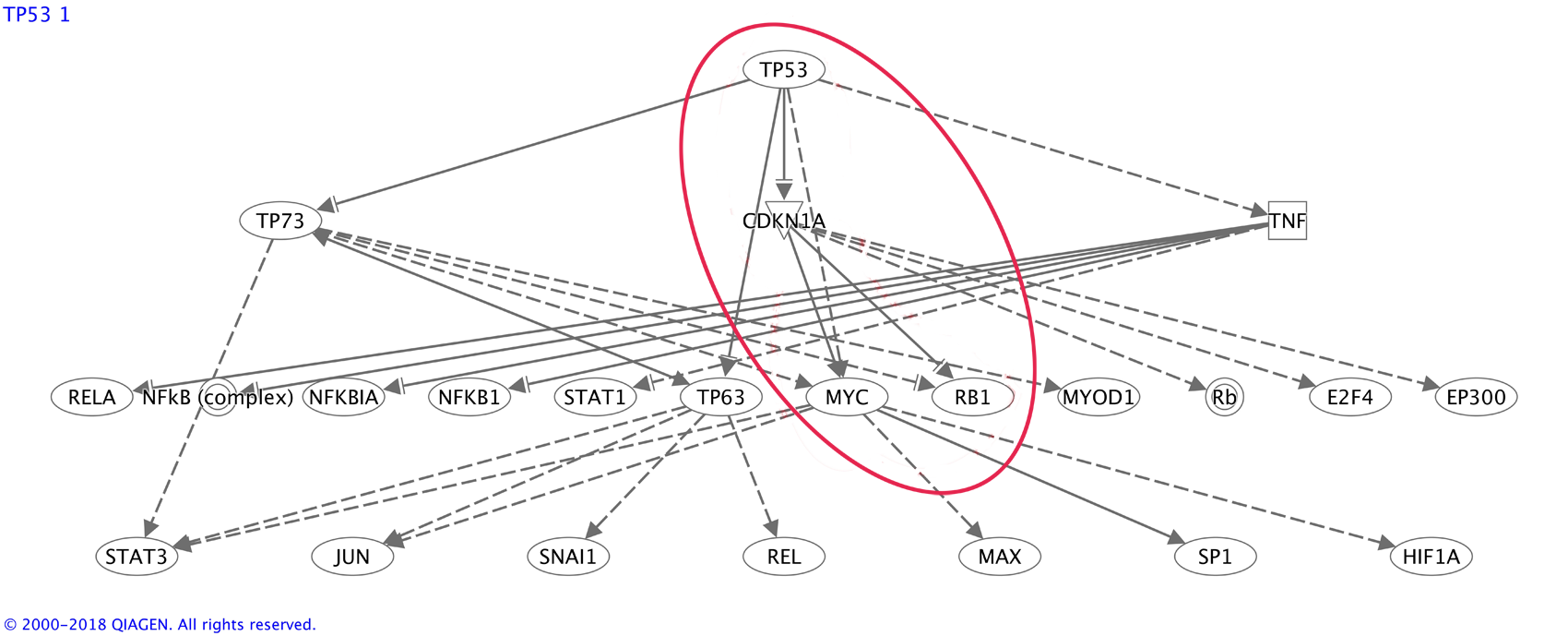

Supplement: Supplementary file 1 [file JCMM-24-1614-s001.TIF]
