# Supplementary material for: Identification of dynamic signatures associated with smoking‐related squamous cell lung cancer and chronic obstructive pulmonary disease
Source: J Cell Mol Med. 2019 Dec 12;24(2):1614–25. doi: 10.1111/jcmm.14852 (PMC6991676; doi:10.1111/jcmm.14852)
Supplement: Supplementary file 2 [file JCMM-24-1614-s002.docx]

Supplementary Material Table 1

The dynamic differentially expressed genes (DEGs) (FDR < 0.01)

| GENEID | GENE_SYMBOL | *p* value of COPD  vs. Normal | *p* value of SQCC + COPD  vs. COPD |
| --- | --- | --- | --- |
| 83853 | ROPN1L | 0.00498934 | 2.89E-25 |
| 80776 | B9D2 | 0.0069219 | 2.58E-24 |
| 5284 | PIGR | 0.005126006 | 9.05E-16 |
| 137075 | CLDN23 | 0.004013307 | 8.14E-14 |
| 55152 | DALRD3 | 0.000382359 | 1.90E-12 |
| 9961 | MVP | 0.002572907 | 8.10E-11 |
| 30815 | ST6GALNAC6 | 0.005388224 | 2.46E-10 |
| 1978 | EIF4EBP1 | 0.007830849 | 3.26E-08 |
| 2744 | GLS | 0.004259369 | 4.01E-08 |
| 2939 | GSTA2 | 0.005057964 | 1.63E-07 |
| 90861 | C16orf34 | 0.006516058 | 4.48E-07 |
| 5699 | PSMB10 | 0.009422407 | 7.45E-07 |
| 629 | CFB | 0.009657903 | 1.15E-06 |
| 1474 | CST6 | 0.006630077 | 1.37E-06 |
| 6342 | SCP2 | 0.008965678 | 1.68E-06 |
| 8763 | CD164 | 0.008623739 | 4.07E-06 |
| 216 | ALDH1A1 | 0.002258029 | 9.07E-06 |
| 2317 | FLNB | 0.001407181 | 1.30E-05 |
| 5447 | POR | 0.009986892 | 2.28E-05 |
| 2817 | GPC1 | 0.002785843 | 3.49E-05 |
| 5355 | PLP2 | 0.004953417 | 3.54E-05 |
| 8190 | MIA | 0.003841795 | 0.000343516 |
| 5971 | RELB | 0.002217147 | 0.000369482 |

| 57007 | CMKOR1 | 0.006687443 | 0.000447435 |
| --- | --- | --- | --- |
| 5411 | PNN | 0.008613559 | 0.000549755 |
| 1153 | CIRBP | 0.004719763 | 0.000901386 |
| 283991 | FAM100B | 0.000733566 | 0.002036827 |
| 9988 | DMTF1 | 0.009342575 | 0.002979483 |
| 29964 | C6orf49 | 0.003063927 | 0.003101513 |
| 8604 | SLC25A12 | 0.008740512 | 0.003331632 |
| 89941 | RHOT2 | 0.002659117 | 0.00408249 |
| 2941 | GSTA4 | 0.007044465 | 0.004315332 |
| 340371 | NRBP2 | 0.009083604 | 0.005869151 |
| 64975 | MRPL41 | 0.009290809 | 0.006333722 |
| 8683 | SFRS9 | 0.008532269 | 0.00688181 |
